# “To exercise sustainably” – Patients’ experiences of compulsive exercise in eating disorders and the Compulsive Exercise Activity Therapy (LEAP) as a treatment: a qualitative interview study

**DOI:** 10.1186/s40337-024-01115-8

**Published:** 2024-10-01

**Authors:** Emma Thell Simón, Elin Monell, Katarina Lindstedt, Anne-Charlotte Wiberg, Emma Forsén Mantilla

**Affiliations:** 1 Department of Neurobiology, Care Sciences and Society, Karolinska Institutet, Stockholm, SE-171 77 Sweden; 2 Stockholm Centre for Eating Disorders, Wollmar Yxkullsgatan 27, Stockholm, SE-118 50 Sweden; 3 Department of Medical Epidemiology and Biostatistics, Karolinska Institutet, Nobels väg 12A, Stockholm, SE-171 77 Sweden; 4 University Health Care Research Center, Faculty of Medicine and Health, Örebro University, Örebro, SE-701 82 Sweden; 5 Centre for Psychiatry Research, Department of Clinical Neuroscience, Karolinska Institutet, & Stockholm Health Care Services, Region Stockholm Norra Stationsgatan 69, Stockholm, SE-113 64 Sweden; 6 Department of Physical Activity and Health, The Swedish School of Sport and Health Sciences, Lidingövägen 1, Box 5626, Stockholm, 114 86 Sweden

**Keywords:** Compulsive exercise, Eating disorder, CBT, Qualitative interviews

## Abstract

**Background:**

Compulsive exercise is common in eating disorders (EDs), but a systematic treatment model is lacking. The CompuLsive Exercise Activity TheraPy (LEAP) is a cognitive behavioral therapy treatment for compulsive exercise in EDs, delivered by trained therapists in groups over four consecutive weeks (8 groupsessions and 1 individual session), aiming to promote healthy physical activity. LEAP is currently evaluated in a randomized efficacy trial. In parallel, it is crucial to learn more about how it is perceived by qualitatively investigating participants’ subjective experiences.

**Methods:**

Nine patients with various EDs participating in the LEAP trial were interviewed about their experiences of taking part in LEAP and about compulsive exercise as an ED symptom using a semi-structured interview guide. The interview transcripts were analyzed according to thematic analysis.

**Results:**

The informants expressed that compulsive exercise had not been addressed in their standard ED treatment and that LEAP as such provided an important complement, spurring reflection, awareness, and changed feelings and behaviors in relation to compulsive exercise. Initially, increased PA was triggered for some, but this side effect was transitory. A wish for more treatment time, in terms of longer or additional sessions, was expressed.

**Conclusions:**

Overall, LEAP seemed to fill an important treatment need and seemed both acceptable and feasible to patients. However, treatment time and the initial increase in PA may need further investigation and attention in order to optimize this treatment.

**Trial registration:**

The trial is registered with the ISRCTN registry (registration date 20200325), trial ID ISRCTN80711391.

**Supplementary Information:**

The online version contains supplementary material available at 10.1186/s40337-024-01115-8.

## Abstract (plain language summary)

Compulsive exercise (CE) is very common in individuals with eating disorders (EDs) often tightly connected with the eating pathology. Even so, most standard treatments do not specifically target CE, leaving patients without strategies to normalize their exercise. The CompuLsive Exercise Activity TheraPy (LEAP) is delivered as an adjunctive treatment to standard ED treatment (targeting CE in patients with EDs. In this study, nine former LEAP patients were interviewed about their experiences of taking part in LEAP and about CE as an ED symptom. The informants were in general satisfied with LEAP and indicated that participation had positive effects on their exercise-related thoughts and attitudes, as well as actual exercise behaviors. They all experienced that CE was not addressed in their standard treatment, although they were motivated to work towards changing it. LEAP was therefore viewed as an important complement. Participating in LEAP initially triggered some informants to exercise more, which fortunately ceased over time and is similar to the temporal negative effect (increased food occupation) often observed initially in cognitive behavioral therapy for eating disorders. The content of LEAP was experienced as valid, informative, and eye-opening, and being able to discuss CE-related topics in a group setting was for many a positive experience. The experiences expressed in this study are very useful for continued development of LEAP. Although preliminary, the results also suggest that LEAP may be a valuable add-on treatment within ED care.

**Supplementary Information:**

The online version contains supplementary material available at 10.1186/s40337-024-01115-8.

## Background

Eating disorders (EDs) are often difficult to treat and about one third of patients remain ill ten years after treatment initiation [1]. Compulsive exercise (CE) is associated with several ED thoughts and behaviors and is often one of the most persistent symptoms [2, 3]. CE is prevalent in about 50% of patients in specialized ED care, regardless of age and gender, and occurs in all ED diagnoses [4]. In patients with anorexia nervosa (AN), CE is associated with higher risk of relapse, more long-term illness, greater emotional suffering, poorer quality of life, and more anxiety and depressive symptoms [5–7]. CE is also associated with longer hospitalizations, greater risk of suicide, and worse treatment outcome in EDs overall [4, 8, 9].

Unfortunately, CE has received less attention in both research and practice compared to other ED behaviors [10]. The traditional approach has often been to ban or strictly restrict physical activity (PA) for individuals in ED treatments due to fear of potential negative effects, including concerns about its presumed negative impact on weight restoration [6, 11, 12]. This approach may however be counterproductive. When individuals with EDs have been interviewed about CE, findings reveal that despite their awareness of its adverse impact on health and the suffering it entails, including pain, exhaustion, and psychosocial consequences, banning PA rather fosters secretive exercise behaviors [13–15]. Further, a growing body of evidence suggests that complete abstinence from PA is neither necessary nor appropriate, as it deprives patients of significant psychological and physical health advantages associated with healthy and adaptive PA [16]. Instead, integrating PA into treatment may in fact facilitate ED treatment and recovery [16, 17]. Patients with EDs also express wanting to be able to engage in PA as a means of self-care to achieve a balanced and healthy lifestyle, but express a clear need for acquiring knowledge about healthy levels of exercise [13, 18]. Furthermore, upon completing treatment, patients have expressed uncertainty regarding what constitutes healthy versus unhealthy exercise, sometimes resulting in a sedentary lifestyle and fear of engaging in PA due to concerns about relapse [15]. This highlights the need to introduce interventions that address CE and promote healthy PA in ED treatment.

However, focusing on CE in treatment has been perceived as challenging for clinicians due to lack of knowledge and absence of general guidelines [10, 19]. For instance, the leading evidence-based treatment for ED, Enhanced Cognitive Behavior Therapy (CBT-E) recommended for all adult patients with ED, lacks specific and targeted interventions for CE [20]. The absence of general guidelines has also hampered development of specific treatment models targeting CE [21, 22]. Despite these challenges, some initiatives to address CE within ED treatment have been undertaken, yielding promising outcomes [2, 12, 17, 23–26]. A recent systematic review of CE treatment approaches in EDs indicates that targeting CE can improve both exercise-related outcomes and eating psychopathology, reduce negative exercise attitudes, and offer benefits across all EDs [17]. The included interventions have supplemented psychoeducation and actual exercise sessions with positive results, however through different approaches [2, 12, 17, 23–26]. Determining which approach works best, for whom, and through which mechanisms (e.g., altered behaviors, thoughts, or emotions) positive change is achieved is therefore still unclear.

Several factors, including psychosocial ones, likely contribute to the development of CE [27]. Meyer and colleagues [3] developed an evidence-based theoretical model focusing the psychological maintenance of CE in EDs. This model highlights how CE may be maintained by several factors, besides the obvious connection to ED desires to lose weight, compensate for food intake, and modify one’s figure. The other factors include compulsivity, perfectionism, rigidity, and emotion regulation, where some or all factors maintain CE in different individuals with EDs. The model outlines how CE for instance may be maintained through false assumptions about exercise, fear of negative consequences (including negative emotions) if exercise is omitted, and difficulties being flexible with exercise routines. The CompuLsive Exercise Activity TheraPy (LEAP) is a CBT-based treatment, originally created by Meyers and colleagues in the UK, with the aim of targeting the maintenance factors in the theoretical model [28]. LEAP is semi-structured and problem-focused with the aim of reducing CE-related attitudes and behaviors and promoting healthy PA. This is mainly done through psychoeducation, discussions, and reflective exercises. There are also some behavioral challenges which aim to help patients modify their exercise behaviors and develop alternative strategies regulating negative affect [29]. This treatment has so far been evaluated with both adult and adolescent (Junior LEAP) patients with AN with tentatively positive results; it seems to decrease ED symptoms and CE, promote more positive attitudes and exercise behaviors, and be regarded as a positive experience when examined qualitatively among adolescents [30, 31]. The effect of LEAP is currently being evaluated in a Swedish randomized multi-center naturalistic efficacy trial involving five ED treatment units. Male and female patients with various EDs reporting CE are included and randomized to either receive LEAP as an adjunctive treatment to their standard ED care or to participate as controls [29].

The aim of the present qualitative study was to investigate patients’ experiences of taking part in LEAP and their experiences of CE as an ED symptom. By interviewing nine former LEAP participants about their experiences of the treatment we aimed to learn more about the patient perspective of LEAP which is crucial for potential implementation and further development. As the study was exploratory the authors did not make hypotheses about potential findings.

### Methods

#### Informants

Informants were LEAP completers in the ongoing Swedish trial [29]. At the time of this study, ten patients at three different treatment units were eligible; nine consented to take part. Eight were females and one was male; three had a diagnosis of AN, two had bulimia nervosa, and four had other specified feeding and eating disorders. Mean age was 25.6 years (range 20–36) and mean illness duration was 8.4 years (range 2–18). In the Swedish trial, LEAP consists of one initial individual session followed by eight 60-minute face-to-face group sessions held twice a week for four consecutive weeks. Outpatients with all diagnoses except binge-eating disorder were eligible if CE was perceived as an issue. All participants had an active standard ED treatment at one of the participating units when included. For more information on the trial, inclusion/exclusion criteria, and treatment design, see study protocol by Monell et al. [29].

#### Instruments

A semi-structured interview guide was co-produced by four of the authors (ETS, EM, KL, EFM; guide provided in Additional file 1). The questions aimed to give a broad picture of the informants’ relationships to PA and what it meant to participate in LEAP. The guide was overseen by a physiotherapist/CBT-therapist with in-depth experience of working with ED treatment and tested with two individuals outside the study setting, both with lived and professional experience of ED with CE. After the test interviews, follow-up questions were added regarding the participants’ relationship to PA, the content in LEAP and what it meant to participate. The final questions covered demographic data, PA prior to and after the ED, the experience of LEAP (structure, content, what it meant to participate), and thoughts about including PA in the treatment in the future (as LEAP currently does not include any PA).

#### Procedure

Participants from the main trial were asked about participating in the present study when contacted for their final assessment (around 12 weeks after LEAP participation). Participants who expressed an interest were then contacted by telephone by the main author who gave further information about the study and the conditions for participating. Informants agreeing to participate provided written informed consent before being scheduled for an interview. Informants could choose between phone or videocall interviews; all chose phone, and the interviews were recorded using the phone recording function. The interviews lasted between 35 and 55 min and were transcribed by the main author. The study was approved by the Swedish Ethical Review Authority (DNR: 2020 − 01173).

#### Analysis

**Fig. 1:**
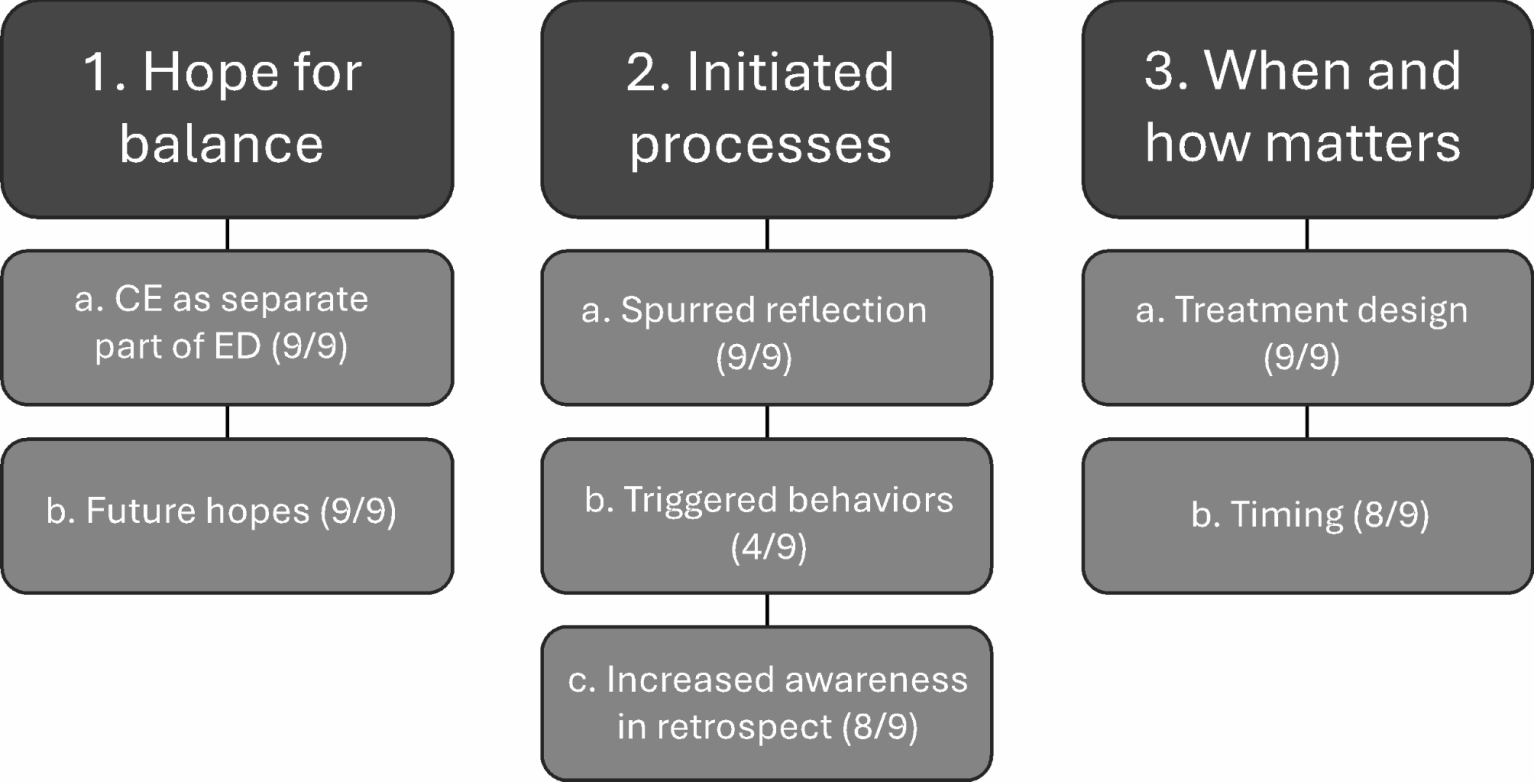
Themes and subthemes. Number of informants represented in each subtheme in parenthesis

The present study is a qualitative, semi-structured interview study with an inductive design. Data was analyzed using Microsoft Word and according to thematic analysis (TA), a theoretically flexible qualitative method [32]. The transcribed data was first evaluated in relation to saturation, that is, when the information obtained is considered sufficient to answer the research questions [33] and when the themes arising are no longer novel. The procedure described by Braun and Clarke [32] was then followed for analyzing the data, using an inductive approach aiming for a combination of latent and semantic themes. This procedure, which includes six steps, was undertaken by the three authors ETS, EM and KL collaborating on the analysis. ETS and KL have extensive experience working with EDs using physiotherapy and psychotherapy, respectively. Both are LEAP-therapists, but not for any of the informants in this study. KL and EM are experienced researchers in the ED field; KL has primarily been conducting qualitative analyses. In the first step - Initital familiarization with data – the three authors read the interviews several times and took notes. In step two and three - Generation of initial codes and Searching for themes – ETS coded the data, sorted the codes and suggested some preliminary themes. In step four and five, the three authors reviewed the suggested themes together, decided on final themes and subthemes, and defined and named themes according to the procedure. In step six - Producing the report – four authors (ETS, EM, KL and EFM) worked together. The analysis conducted resulted in three main themes and seven sub-themes (Fig. 1).

## Results

The analysis resulted in three main themes: **(1) Hope for balance**, **(2) Initiated processes** and **(3) When and how matters** (see Fig. 1). The first theme described how the ED influences exercise behaviors including thoughts about future PA and was further subdivided in: *(a) CE as separate part of ED* and *(b) Future hopes*. The second theme described LEAP participation related to insights, cognitions and behaviors and constituted three sub-themes: *(a) Spurred reflection*, *(b) Triggered behaviors*, and *(c) Increased awareness in retrospect*. The third theme focused when and how LEAP ought to be delivered and was subdivided into *(a) Treatment design*, and *(b) Timing*.

### Hope for balance

This theme describes how informants experienced a change in their relation to PA as they developed an ED, their experience of PA while in ED treatment, and their thoughts about PA in the future.

#### CE as separate part of ED

All informants had been physically active at different levels from a young age. However, as they developed an ED, activity that had previously been joyful became a rigid, monotonous, and compulsive way of burning calories, regulate negative affect, compensate for food intake, and manage thoughts about weight and shape. It also became a way of maintaining a certain view of oneself, and a strive towards ideals. Some individuals differentiated between exercise that they engaged in alone and competitive sports performed with others, with the former perceived as more compulsive and problematic.

*“It has been a long time since exercise was fun and joyful. It (PA) has many functions - reducing anxiety*,*controlling weight*,* performing*,* to show off as someone who exercises*,*a lot to do with the body*,* the self-image and ideals”.* [6]

Some experienced that their PA levels had varied over time. When at its worse, many were active in different ways for hours a day. Resting made them anxious and exercise was prioritized above other things in life and carried out despite injury or illness.

*“When at its worst*,* so to speak*,* it was seven sessions per week*,* every day. Sometimes it was double the amount*,* but usually probably two-three hours. On top of that*,* I walked for about three-four hours a day. If I rested for one day I felt really anxious*,* or if I ate too much according to myself… I had to compensate even more the next day”.* [2]

The informants were frustrated that their CE had not been given attention or been fully addressed in treatment, despite their experience of CE being such a central part of their ED. CE was identified by therapists, and they were encouraged to reduce their activity, but without further support on how to accomplish such change. Some individuals had been prohibited from exercising but received no support when they eventually were allowed to be active again.

*”When I have brought it up (the exercise) the answer has basically been “yes but you have to abstain from exercising now*,* but you don’t have to do that forever”*,* something like that*,* and then we haven’t really talked about it anymore”.* [8]

CE was also experienced as driven by several motives, not just weight loss, though ordinary treatment primarily focused on the latter.

*“It sometimes felt like*,* earlier on (in treatment) that there has been a lot of focus on that all exercise is bad for you. Only driven by a desire to lose weight. I didn´t experience it like that. You get into an exercise routine you neither want nor have the energy to do*,* nor enjoy. If I exercised this much*,* then I had to do at least the same amount next time. then*,* it easily becomes a vicious circle. where the exercise becomes time consuming and creates anxiety rather than something positive. And you feel obliged and exercise to avoid getting bad conscience”* [9].

Many expressed a wish to regain an enjoyable and healthy relationship to PA. LEAP was experienced as beneficial to gain both knowledge and strategies useful in everyday life, and many appreciated the possibility to reflect upon one’s exercise behaviors.

*“Since I have experienced for years that my exercise…loses its purpose for me*,* that I want exercise to be fun*,* I want to be able to exercise and actually feel good as a result. And I saw this (LEAP) as an opportunity for me to perhaps rediscover this wishand this kind of healthy thinking and relation to exercise.”* [1].

#### Future hopes

All informants expressed that they wanted to live an active life with enjoyable and balanced PA. As a result of participating in LEAP, they also expressed hope to in the future be able to engage in joyful exercise, treat themselves with more kindness, and rest without feeling anxious. To exercise sustainably, they planned on having a more low-key, sensible attitude towards exercise, with reasonable goals, more in tune with their bodily signals and greater awareness of ED thoughts.

*“I see that I have hope…I see that I will try to recognize my boundaries and be able to prioritize other things when I need to*,* that it (exercise) should not rule my everyday life.”* [6].

*               “I don’t want it (exercise) to feel like an obligation. If you skip training at some point or if you’re ill and can’t train that much for a few days*,* that it shouldn’t be an inner stress and something that causes anxiety and feels difficult. So that’s my hope*,* for it to be a balance.”* [5].

Many also described a future hope for being more flexible in relation to their PA: *“to not be a slave under it”* [8], to avoid letting PA being prioritized above other important areas in life such as studies, relationships or work.

*“If there is too much of it*,* performance*,* in exercise*,* it’s not fun at all and I try to focus more on sustainability over time”* [9].

Several informants talked about the risk of relapsing into using PA as their way of coping, for instance with difficult emotions, and stressed the importance of receiving an ED treatment where they work with all parts of their illness (i.e., including CE) to be able to: *“exercise to feel good*,* not to avoid feeling bad”* [3]. As a result of participating in LEAP, they also had hopes of being able to manage potential set-back through strategies and long-term goals, but also by being open about their difficulties and accepting support from loved ones.

*“I hope I can handle this*,* because I constantly return to thoughts like: “what is important to me” and “what do I prioritize”*,* “what do I want in the long run”*,* and that I can have people around me who knows about my background*,* so that I will always dare to say that it’s difficult right now.”* [2].

### Initiated processes

This theme describes what LEAP initiated in terms of thoughts, behaviors, and changed awareness.

#### Spurred reflection

LEAP seemed to stimulate thoughts, different perspectives, and engaging reflections. The content of the sessions was described as “interesting”; understanding and discussing the mechanisms maintaining CE was described as “thought provoking”. The informants experienced the fact-based content as relevant and relatable to their difficulties and their own experiences.

*“I had different ideas that I believed in about how much one should exercise and about eating in a certain way and all sorts of things*,* and it lead to a situation that eventually became dangerous. To be shown these kinds of different models and try to understand*,* and then start challenging it to realize that it is not dangerous to rest*,* that nothing super drastic will happen.”* [5].

LEAP also acknowledged that CE is driven by more aspects than a drive for thinness. *“You get some sort of confirmation that you have other problems (in exercise) than “exercise to lose weight”. It was accepted. There are so many more aspects of exercise that can be negative and difficult when you have an ED”.* [9]

Several informants expressed that LEAP provided them with a lot of new information. Some recognized CBT-techniques from their standard treatment but found LEAP more focused on PA. The knowledge and discussions about the factors maintaining CE were novel to most and the activities in relation to these were often experienced as eye-opening.

*              I found it very interesting and very good with these different maintaining factors*,* and especially that a lot of time was spent going through all of them. Because that’s probably what I have experienced as most helpful for me in hindsight*,* that I have gained an understanding that there are several things that keep it going. It’s not just that I have an ED but it can also be due to the fact that I am compulsive or that I am very difficult to let go of my routines*,* or such things. And I haven’t thought about that at all before*,* what keeps the behavior going*,* you know.”* [7].

Sharing experiences with others in the group was highly valued. It gave a sense of connection, of not being alone with these difficulties, and of being seen. Individual insights about one’s relationship with PA also increased through group discussions.

*“My goals that I have had previously*,* they are not reasonable*,* it is not strange that I have not reached them”.* [4]

Many informants were surprised that the group discussions were rewarding and experienced as quite easygoing. This spurred reflection even though the group participants had different experiences or opinions.

*”It was more fun and sometimes easier to bounce thoughts around specific things related to our EDs even though we may not necessarily agree with each other all the time. It felt more productive at times to have group discussions about such sensitive topics.”* [1].

#### Triggered behaviors

Four informants expressed that LEAP initially triggered an increase in PA because of feeling more anxious, comparing oneself to others in the group, and a sense of having to really delve into it now, to then be able to leave it behind.

*“It has been somewhat triggering in a way to hear everyone else’s issues and the feeling that I wasn’t sick enough. At first*,* it became more triggering before I kind of came to the realization that yes*,* but I am also ill*,* but my illness looks different”.* [3]

Informants described how this increase in PA started to decline after three to four sessions of LEAP. At that time, they felt more comfortable in the group and dared to talk more openly about their experiences of being triggered. This resulted in a sense of a “new beginning” with a calmer attitude towards PA. Some informants raised that the therapists could have informed them already in the beginning about the potential of triggering processes and social comparison, to reduce their anxiety.

While the group climate could trigger comparison, many felt that it was positive that their difficulties manifested differently within the group, challenging their image of both CE and ED. Just like the initial increase in PA, the comparison also decreased when informants started to feel more comfortable with each other.

#### Increased awareness in retrospect

Most informants experienced a change in their thoughts and attitudes towards PA a few months after completing LEAP. They described this change as being able to take a step back, understand reactions in their bodies, reflect upon emotions and thoughts, and have a more long-term focus rather than reacting in the moment.

*“I actually felt that*,* when I was there*,* that it (LEAP) did not give me that much. But then in the end I could see*,* sort of*,* that it actually did somehow change my point of view or way of thinking. And that is probably due to the treatment*,* helping one to discuss and take a step back and observe it.”* [6].

Many had also changed their pattern of PA after LEAP, for instance by adding recovery days, taking breaks during training sessions, and lowering their own demands. They also experienced more joy in their activity and had managed to keep it at a less intense level. Learning more about emotion regulation in relation to PA and being able to stay in an emotion and accept it, was mentioned as helpful and created an enhanced awareness about emotions in relation to PA. Several informants highlighted that they had become aware of their own reliance on exercise as a means for regulating negative affect and that this dependence felt unsustainable.

*“That dynamic is not fair to me*,* being dependent on exercise [to decrease anxiety].”* [1].

*“Later*,* when the treatment was completed*,* you may realize that many things have been related to earlier stuff and so on*,* that it is put in a context. To acquire tools to deal with your emotions in relation to exercise and to stay in it and learn has resulted in me not having the sort of emotional breakdowns in relation to physical activity as often.”* [6].

Several informants expressed that ED thoughts and urges to be active remained, though after LEAP they found it easier to resist such urges. They expressed that LEAP had increased their awareness of how their activity was governed by rules and that this opened for a possibility to start reducing the control and attempt to engage in recovery and joyful exercise, for increased well-being. LEAP also seemed to increase the informants’ awareness about the function of PA and the importance of listening to bodily signals, even though that was experienced as immensely difficult.

*“By and large*,* it [LEAP] has changed my relationship to exercise. Exercise should still be something I can enjoy*,* something I like because that is really why I work out. I have now calmed down*,* I try to see it from a different perspective*,* what is my body capable of now*,* and I tune in much more. So I am probably a bit more accepting*,* even though I think this acceptance is one of the hardest things.”* [1].

Many informants experienced LEAP as valuable and useful, and added that they see a need for it to be further developed and become more widespread.

*             ” I needed these strategies to be able to exercise in a healthier way. I wouldn’t have been able to start exercising if I hadn’t joined LEAP*,* I think I had remained in the worse condition. I think it’s really important with LEAP. It needs to start on a large scale [within ED care].”* [6].

### When and how matters

This theme describes the structure of LEAP and when LEAP should be conducted during ED treatment time.

#### Treatment design

The informants experienced LEAP and its components as concrete, informative, and leading to results with no content missing. Most experienced the sessions of 60 min as too short and wished to double the time to finish tasks and have time for more in-depth discussions of the topics/subjects.

*“I think they covered good topics*,* but then these sessions were so short*,* it was only an hour*,* so we sort of never had the time to really go in deep.”* [5].

The behavioral experiment was experienced as a valuable concept, that the informants were happy to continue to work with after LEAP. However, more time and support would have been appreciated and beneficial during the sessions.

*           ” I found it difficult to come up with a challenge that was just right and not too easy and then “is the purpose to be able to take the bus or is the purpose to feel that it’s okay to take the bus?”. That there are many steps involved. And that we didn´t really have time to talk it through.”* [7].

Everybody participated in small groups with two, four or five participants, and all agreed that a small group was preferable for everybody to be able to speak up. However, some thought that two participants were too few to share perspectives. Regardless of the number of group participants, the time per session was perceived as scanty, but in the end, when the intervention gained context, the treatment was experienced as extensive enough. Some reflected that although longer sessions could potentially be helpful, they might end up being tiring. Shorter sessions might also facilitate participation.

*“It might be easier to implement change if it is more compressed. And if you do other things*,* like studying*,* it might be harder to make time for things that last a long time”.* [9]

A couple of informants wished for LEAP to be spread out over a longer period by having sessions once a week, to have more time to apply techniques between sessions. *“You would try to apply it to yourself*,* but it felt like you didn’t quite have time”* [5]. However, there were perceived advantages of an intensive treatment, as the informants did not lose focus in between sessions or had to wait too long if they felt they needed assistance. As an alternative to extending LEAP, a booster session was suggested two weeks after the intervention, to for instance follow-up on the behavioral challenges.

*“Of course*,* there are pros and cons. If the sessions are too far between*,* you kind of have time to forget. That’s the advantage of having it a little more intensive*,* that you really keep momentum in the process*,* and that you can also get help if you get stuck.”* [8].

It was perceived as a good balance that the group was led by a psychologist and a physiotherapist, the latter to provide more specific knowledge about PA. Several informants would have liked individual advice about PA. Most, but not all, were further positive towards complementing LEAP with ​​PA in a clinician-led group, as a potential opportunity to learn more about and to resume healthy and adequate PA, preferably after weight recovery. Such PA was believed to potentially contribute to a shift of focus and improved body awareness and acceptance: *“To shift the focus from it being exercise*,* to maybe learn to listen to the body in some way”* [3]. The informants did see a risk of comparison, but most thought positive group effects, such as a sense of community, would outweigh that risk, given that participation was voluntary.

#### Timing

The informants had been in their standard treatment for at least three to four months prior to LEAP, but most around six months. All agreed that LEAP seems best suited in the final phase of standard treatment, after having gained certain insights, a more flexible mindset, feeling more comfortable in the group setting and being more receptive to change. *“If I had been in the LEAP-study at the very beginning of my treatment*,* I don’t think I would have gotten these insights.”* [1].

With earlier LEAP initiation, some speculated that the overall ED issues might have overshadowed the specific CE focus. However, others found it potentially helpful earlier on, especially when PA was tightly connected to their mindset around food. Even so, many thought that LEAP fitted better later in treatment when everyday life had begun to normalize, to be better able to apply strategies, home tasks, and behavioral challenges. It also seemed beneficial to engage in some kind of PA during LEAP, as it otherwise can be difficult to remember thoughts and feelings connected with PA.

*“I would have been a little bit more helped by it [LEAP] even a little further on*,* now that I feel that*,* as I said*,* I am a little more flexible. If you haven’t exercised for a very long time*,* you might not really remember exactly how you thought and felt when you did exercise.”* [5].

LEAP also matched the final phase of standard treatment when leisure interests are usually integrated, as PA had been a leisure interest for most since a young age. It also felt natural to end ED treatment with LEAP “*to then sort of be. set free.”* [4].

LEAP was perceived as a helpful complement to standard ED treatment and vice versa, although having both in parallel could be somewhat intense. There were however requests for LEAP to be better integrated with the standard treatment, by having a focus on PA also in standard treatment during and after the LEAP period.

*“So you don’t forget*,* you might need more support to apply it according to your own needs. That the therapist has it (PA) as a part of your treatment plan*,* to follow up.”* [9].

### Potential improvements of LEAP through the patients’ perspective

Aggregated potential improvements to LEAP found in the accounts of the informants are summarized below in Table 1.

**Table 1:** Potential improvements of LEAP

1.	There will be individual variations related to when LEAP is most suitably initiated in relation to the standard treatment, but the final phase seems preferrable.
2.	Clinicians need to inform patients that there might be an initial negative, yet transitory, effect on CE.
3.	Clinicians also need to address social comparison early on.
4.	Evaluate whether session time could be slightly extended.
5.	Increase support around the behavioral challenge and spend more time focusing this during the sessions.
6.	Allocate plenty of session time for discussions.
7.	Evaluate whether LEAP can be delivered less frequently (i.e., one session/week), alternatively whether a booster session a few weeks post LEAP could be added.
8.	Make LEAP more integrated with standard treatment by assuring a temporary special focus on PA and CE also in standard treatment during LEAP.
9.	Groups should preferably be held by a physiotherapist and a psychologist/psychotherapist.
10.	Evaluate if safe, guided PA could be added as a complement.

CE = compulsive exercise; LEAP = CompuLsive Exercise Activity TheraPy; PA = physical activity

## Discussion

The aim of this qualitative study was to investigate how patients with ED and concurrent CE experienced the CBT-treatment LEAP. Overall, the informants were satisfied with LEAP and indicated positive effects on CE-related thoughts, attitudes, and behaviors after some time. A general finding was the experience that CE was not addressed in their standard treatment, although all reported being motivated to work towards changing their CE attitudes and behaviors. In the initial sessions, some informants were triggered to exercise more which fortunately ceased over time. The content was experienced as valid and informative, though time constraints sometimes resulted in discussions and activities being cut short. LEAP was seen as a helpful complement preferably added towards the end of a standard treatment period.

### Hope for balance

Exercise was central in most informants’ lives and in their EDs, where LEAP offered a wider perspective on the motives for engaging in CE. The informants had been physically active before developing an ED, but the ED changed the quality of their activity to become more of a monotonous, rigid compulsion, with the aim of both controlling weight and shape, and regulate affect. Previous functional exercise has been identified as a risk factor for engaging in CE when developing an ED [34, 35] and the mentioned transformation in quality have also been reported by others [27], as well as described by the underlying theory of CE maintenance [3].

The informants experienced a lack of focus on CE in their standardized treatment, a deficit confirmed in other studies [10, 13]. This may be related to a lack of clinical guidelines and knowledge about CE [22], and fear of potential negative effects of working with PA in this population [6]. However, the common approach of prohibiting or strongly restricting PA in this patient group may be problematic as it could increase anxiety and depressive symptoms, requires intense monitoring, fosters secrecy, and may affect treatment alliance negatively [13–15, 19]. The informants wished to find a joyous and healthy way of relating to PA and just like in previous studies [13], the need for working towards normalizing PA rather than prohibiting it, was highlighted. Resorting to exercise as a coping strategy during hard times was a risk mentioned in this study and as CE also is linked to higher rates of relapse [5], this further elucidates the need to address CE in treatment.

### Initiated processes

LEAP increased the informants’ knowledge about CE, which in turn spurred reflection, also in retrospect. This is valuable in terms of widening patients’ perspectives [10]. Insights about healthy versus unhealthy exercise were gained and patients’ preconceptions about what constitutes healthy exercise were challenged. Learning more about factors maintaining CE was experienced as eye-opening for many and this is in line with previous results [17, 31]. Informants reported wanting to be less compulsive, rigid, and in particular, less reliant on CE for emotion regulation [3]. This new knowledge and insights seemed to prepare the foundation for actual changes in thoughts, attitudes, and behavioral strategies, and gradually being comfortable with less CE. The positive changes seemed to come about one to three months post the treatment. Similarly, the effects of CBT-E tend to continue post treatment as the patient continues to apply newfound knowledge, skills, and strategies [20], thus an unsurprising result.

However, what seemed most central for several informants was the kinship and feelings of closeness in the group. This differs from the study focusing on Junior LEAP, in which the increased knowledge was most appreciated [31]. This may suggest adults have different needs; for instance, several of our informants felt they already had some knowledge beforehand. Initially the group format also contributed to negative social comparison, although the variability between patients in the groups was also mentioned as positive. The tendency to compare oneself to others in the group ceased over time but was mentioned as something the therapists could have highlighted at the start of the group. Adolescents with AN taking part in a group intervention related to PA described something similar; the group was helpful but initially triggered comparison and competitive thoughts and behaviors, in parallel [13].

About half of the informants also experienced that LEAP initially triggered increased PA, perhaps as a result of both social comparison and the dedicated focus on PA that comes with LEAP. This response seemed to subside after about three sessions and is similar to the temporal negative effect often observed in CBT-E, where patients may be more occupied by food initially in treatment [36]. The initial increase in PA has not been reported previously, but this may have more to do with the scarcity of research in CE interventions, including qualitative, than a specific flaw of LEAP. The informants suggested that therapists should bring up the topic of initial triggering of PA at the start of the treatment, which is also the recommendation in CBT-E [20].

### When and how matters

The content in LEAP was experienced as “to the point” and “informative” and there was no mentioning of missing topics, equivalent to the previous study of Junior LEAP [31]. The one-hour long sessions were however sometimes experienced as stressful. This could be an effect of these patients being recruited from the first LEAP-groups held, where the therapists were new to the manual, time allocation, and the session activities. The LEAP manual is semi-structured with several suggested session activities, which may be omitted if group discussions are fruitful. However, as this was the first time the therapists delivered LEAP, they potentially aimed to adhere to the manual rather strictly, which could have resulted in activities being rushed and/or discussions being cut short. There was a wish for more in-depth focus on some subjects and more time devoted to the behavioral challenge as it was deemed important for behavioral change. This may also be an effect of the above-mentioned lack of focus on CE in their ordinary treatment. A few informants also expressed a wish for having more time between sessions to complete homework and work with their behavioral challenge, or alternatively add booster sessions. This was also expressed in another study focusing on an intervention for CE [24] and in general, booster sessions seem valuable to prevent ED relapse [37, 38].

Mean illness duration for these informants was 8.4 years, suggesting that LEAP may be experienced helpful despite long-term illness. However, many would have liked something like LEAP earlier on in their illness courses. Prior research indicate that CE needs to be addressed early on to potentially decrease the risk of more long-term illness duration [6], and as such, to a worse overall prognosis [39]. LEAP was considered a good complement preferably towards the end of standard ED treatment, although optimal timing may differ between individuals and therefore should be individually assessed. Other studies focusing on interventions for CE have included individuals that have started to regain weight, thus being some time into their standard treatment. However, in this study patients did not necessarily need to regain weight, but there could be other benefits from adding LEAP later on; they may have gained some insights, knowledge, and skills in relation to their eating pattern that may be helpful also when focusing their CE. They may also be less prone to negative processes such as comparison further along in treatment.

Lastly, most felt positive about the idea of receiving group-based guided PA as a complement to LEAP, to learn more about bodily signals and get hands-on experience of healthy exercise. Previous studies show positive effects of well-designed and individually tailored PA interventions with ED patients, for instance in terms of more positive body-image, higher quality of life and decreases in ED symptoms, anxiety, and depressive symptoms [16, 17, 40, 41]. Future studies may therefore explore if and how a component of safe, guided PA could be added to LEAP.

### Clinical implications

The patient perspective is of great importance for new treatments. As such, these results are valuable for the implementation and further development of LEAP as well as informative for other treatments systematically focusing CE. It is evident that addressing CE in standard ED treatment is not consistently considered. Instead of simply banning PA as the main intervention, regardless of somatic status, exercise needs to be approached and normalized [13]. Working with the factors maintaining the problematic exercise behavior earlier on in treatment may prevent a more long-term prognosis and potential relapses [5], which was also expressed by some informants. The LEAP-specific experiences expressed in this study are of utmost importance as a means for continued development of LEAP. The promising results suggest that LEAP may be an add-on treatment to consider offering patients more large-scale within ED care, but the results of the ongoing RCT are needed to give more guidance and certainty. The group format was appreciated, and the fact that physiotherapists led the groups (together with psychologists) is also a natural way of integrating a greater focus on PA in ED treatment. The informants gave helpful suggestions for improvements where some might be implemented straight away by the therapists delivering LEAP in its current format. This include for instance informing about CE and social comparison potentially being triggered initially, greater focus on the behavioral challenge, and allocation of as much time as possible for group discussions. Other suggestions, such as changes in session time and inclusion of PA in group are valuable for further LEAP development.

### Strengths and limitations

A strength of using a semi-structured interview guide is that important areas are covered while also allowing informants to speak freely [33]. TA is a well-known and often applied method of analysis within psychology as it results in a rich and detailed material [32]. Several authors were involved in coding the material into themes allowing for reflexivity and a breath of interpretations [42]. Reliability was further strengthened by the fact that saturation in the analysis was reached [33]. Internal validity may be considered a weakness as none of the informants read the transcribed interviews in retrospect, however three of the authors read the transcriptions and agreed with the identified themes. Our informants belonged to the first wave of LEAP groups in Sweden. This may impact our results as the clinicians had no previous experience of delivering LEAP, which could for instance affect session-planning as mentioned above. The time passed between completing LEAP and being interviewed could have impacted the informants’ experiences, especially as most continued receiving standard ED treatment. Retrospective insights (potentially gained via bringing up CE-related issues in the standard treatment after completing LEAP) are nevertheless important, and as the design of the main LEAP trial is naturalistic (i.e. standard treatment is intended to continue after patients finish LEAP), such synergy effects are not disadvantageous. LEAP is a short intervention, and similarly to e.g. CBT-E [20] its effects are likely to continue after completing it, which is also why the interviews were conducted about 3 months after completing LEAP. Finally, not conducting the interviews face-to face may have been a disadvantage for some. On the other hand, conducting the interviews by phone made participation convenient and the fact that they could choose between phone or videocall also gave the informants some control over the situation.

### Future research

Several aspects were raised that require further study. The lack of focus on PA and CE in standard ED treatment suggests we need to learn more about the knowledge and experiences of clinicians working with EDs. The initial triggering of PA and social comparison could be investigated further in terms of how common this is for patients and how negative effects can be minimized. The structure of the intervention (e.g. length of sessions, timing in relation to standardized treatment and whether booster sessions amplify potential treatment gains) needs further investigation to better tailor it to patients’ needs. Evaluating the potential of combining LEAP and well-controlled clinician-led PA in group is also a future venue, as well as working towards evidence-based guidelines on how to best assess, address, and treat CE in this patient group.

## Conclusions

CE is a common symptom in all ED diagnoses and should therefore be addressed as part of treatment. Lack of guidelines, knowledge, and fear of adverse effects have hampered CE treatment development.There are however promising efforts, including the CompuLsive Exercise Activity TheraPy (LEAP), a CBT group treatment aimed at reducing CE-related attitudes and behaviors and promoting healthy PA. In this study, exploring the patient perspective, informants expressed that LEAP accurately met an overseen need, ignited hope of finding balanced and joyous PA, provided helpful and thought-provoking knowledge about CE, and positively changed CE related thoughts, attitudes, and behaviors. More knowledge about LEAP as well as CE in general is needed, but overall, LEAP seems to be a promising treatment potentially filling the CE related treatment gap.

## Electronic supplementary material

Below is the link to the electronic supplementary material.

Supplementary Material 1: Additional file 1.docx contains “Additional materials to: “To exercise sustainably” – Patients’ experiences of compulsive exercise in eating disorders and LEAP as treatment: a qualitative interview study. The interview guide”, which is the semi-structured interview guide used in this study.

## Data Availability

No datasets were generated or analysed during the current study.

## Abbreviations

AN: Anorexia Nervosa
CBT: Cognitive Behavioral Therapy
CBT-E: Enhanced Cognitive Behavioral Therapy
CE: Compulsive Exercise
ED: Eating Disorder
LEAP: The Compulsive Exercise Activity TheraPy
PA: Physical Activity
RCT: Randomized Controlled Trial
TA: Thematic Analysis

## References

[1] Eddy KT, Tabri N, Thomas JJ, Murray HB, Keshaviah A, Hastings E, et al. Recovery from Anorexia Nervosa and Bulimia Nervosa at 22-Year Follow-Up. J Clin Psychiatry. 2017;78(2):184–9. 28002660 10.4088/JCP.15m10393 PMC7883487

[2] Calogero RM, Pedrotty KN. The practice and process of healthy exercise: an investigation of the treatment of exercise abuse in women with eating disorders. Eat Disord. 2004;12(4):273–91. 16864521 10.1080/10640260490521352

[3] Meyer C, Taranis L, Goodwin H, Haycraft E. Compulsive exercise and eating disorders. Eur Eat Disord Rev. 2011;19(3):174–89. 21584911 10.1002/erv.1122

[4] Monell E, Levallius J, Forsén Mantilla E, Birgegård A. Running on empty - a nationwide large-scale examination of compulsive exercise in eating disorders. J Eat Disord. 2018;6:11. 29942510 10.1186/s40337-018-0197-z PMC5996558

[5] Carter JC, Blackmore E, Sutandar-Pinnock K, Woodside DB. Relapse in anorexia nervosa: a survival analysis. Psychol Med. 2004;34(4):671–9. 15099421 10.1017/S0033291703001168

[6] Strober M, Freeman R, Morrell W. The long-term course of severe anorexia nervosa in adolescents: survival analysis of recovery, relapse, and outcome predictors over 10–15 years in a prospective study. Int J Eat Disord. 1997;22(4):339–60. 9356884 10.1002/(sici)1098-108x(199712)22:4<339::aid-eat1>3.0.co;2-n

[7] Young S, Touyz S, Meyer C, Arcelus J, Rhodes P, Madden S, et al. Relationships between compulsive exercise, quality of life, psychological distress and motivation to change in adults with anorexia nervosa. J Eat Disord. 2018;6:2. 29441204 10.1186/s40337-018-0188-0 PMC5799909

[8] Smith AR, Fink EL, Anestis MD, Ribeiro JD, Gordon KH, Davis H, et al. Exercise caution: over-exercise is associated with suicidality among individuals with disordered eating. Psychiatry Res. 2013;206(2–3):246–55. 23219104 10.1016/j.psychres.2012.11.004 PMC5558595

[9] Solenberger SE. Exercise and eating disorders: a 3-year inpatient hospital record analysis. Eat Behav. 2001;2(2):151–68. 15001043 10.1016/s1471-0153(01)00026-5

[10] Quesnel DA, Libben M, N DO, Willis-Stewart MIC, Caperchione S. Is abstinence really the best option? Exploring the role of exercise in the treatment and management of eating disorders. Eat Disord. 2018;26(3):290–310. 29131718 10.1080/10640266.2017.1397421

[11] Davies S, Parekh K, Etelapaa K, Wood D, Jaffa T. The inpatient management of physical activity in young people with anorexia nervosa. Eur Eat Disord Rev. 2008;16(5):334–40. 18059074 10.1002/erv.847

[12] Hausenblas HA, Cook BJ, Chittester NI. Can exercise treat eating disorders? Exerc Sport Sci Rev. 2008;36(1):43–7. 18156953 10.1097/jes.0b013e31815e4040

[13] Chubbs-Payne A, Lee J, Isserlin L, Norris ML, Spettigue W, Spence K, et al. Attitudes toward physical activity as a treatment component for adolescents with anorexia nervosa: an exploratory qualitative study of patient perceptions. Int J Eat Disord. 2021;54(3):336–45. 33185901 10.1002/eat.23411

[14] Kolnes LJ. Feelings stronger than reason’: conflicting experiences of exercise in women with anorexia nervosa. J Eat Disord. 2016;4:6. 26962455 10.1186/s40337-016-0100-8 PMC4784414

[15] Moola FJ, Gairdner S, Amara C. Speaking on behalf of the body and activity: investigating the activity experiences of Canadian women living with anorexia nervosa. Ment Health Phys Act. 2015;8:44–55.

[16] Mathisen TF, Sundgot-Borgen J, Bulik CM, Bratland-Sanda S. The neurostructural and neurocognitive effects of physical activity: a potential benefit to promote eating disorder recovery. Int J Eat Disord. 2021;54(10):1766–70. 34259338 10.1002/eat.23582

[17] Hallward L, Di Marino A, Duncan LR. A systematic review of treatment approaches for compulsive exercise among individuals with eating disorders. Eat Disord. 2022;30(4):411–36. 34029170 10.1080/10640266.2021.1895509

[18] Brunet J, Del Duchetto F, Wurz A. Physical activity behaviors and attitudes among women with an eating disorder: a qualitative study. J Eat Disord. 2021;9(1):20. 33568228 10.1186/s40337-021-00377-w PMC7877068

[19] Davies RR. The treatment of compulsive physical activity in anorexia nervosa lacks a conceptual base. Volume 3. Adv Eat Disord; 2015. pp. 103–12. (Abingdon, U K). 1.

[20] Fairburn CG. Cognitive behavior therapy and eating disorders. New York: Guilford Press; 2008.

[21] Noetel M, Dawson L, Hay P, Touyz S. The assessment and treatment of unhealthy exercise in adolescents with anorexia nervosa: a Delphi study to synthesize clinical knowledge. Int J Eat Disord. 2017;50(4):378–88. 28093835 10.1002/eat.22657

[22] Quesnel DA. Evaluating the impact of a safe Exercise Training Workshop on Knowledge and Self-Efficacy to Manage Dysfunctional Exercise among eating disorders clinicians at Alsana Eating Disorders Center. Ontario: The University of Western Ontario; 2022.

[23] Danielsen M, Rø Ø, Romild U, Bjørnelv S. Impact of female adult eating disorder inpatients’ attitudes to compulsive exercise on outcome at discharge and follow-up. J Eat Disord. 2016;4:7. 26966516 10.1186/s40337-016-0096-0 PMC4785623

[24] Dittmer N, Voderholzer U, Mönch C, Cuntz U, Jacobi C, Schlegl S. Efficacy of a Specialized Group Intervention for Compulsive Exercise in inpatients with Anorexia Nervosa: a Randomized Controlled Trial. Psychother Psychosom. 2020;89(3):161–73. 32036375 10.1159/000504583

[25] Quiles Marcos Y, León Zarceño E, López López JA. Effectiveness of exercise-based interventions in patients with anorexia nervosa: a systematic review. Eur Eat Disord Rev. 2021;29(1):3–19.

[26] Toutain M, Gauthier A, Leconte P. Exercise therapy in the treatment of anorexia nervosa: its effects depending on the type of physical exercise-A systematic review. Front Psychiatry. 2022;13:939856. 36339831 10.3389/fpsyt.2022.939856 PMC9627498

[27] Kolar DR, Gorrell S. A call to experimentally study acute affect-regulation mechanisms specific to driven exercise in eating disorders. Int J Eat Disord. 2020; 54(3):280-86. 10.1002/eat.23427 PMC8166956 33289120

[28] Taranis L, Touyz S, La Puma M, Meyer C. Compulsive Exercise Activity Therapy: Group Cognitive Behavioral Therapy for patients with compulsive exercise and eating disorders. Therapist Manual. Swedish version: Forsén Mantilla E, Monell E, Clinton D. Stockholm: Karolinska Institute; 2017. 2011. Loughborough: Loughborough University.

[29] Monell E, Meyer C, Szwajda A, Forsén Mantilla E. Taking the LEAP: study protocol for a randomized, multicentre, naturalistic, efficacy trial of the compuLsive Exercise Activity theraPy (LEAP) - a cognitive behavioral program specifically targeting compulsive exercise in patients with eating disorders. BMC Psychiatry. 2021;21(1):369. 34301226 10.1186/s12888-021-03356-2 PMC8299452

[30] Hay P, Touyz S, Arcelus J, Pike K, Attia E, Crosby RD, et al. A randomized controlled trial of the compuLsive Exercise Activity TheraPy (LEAP): a new approach to compulsive exercise in anorexia nervosa. Int J Eat Disord. 2018;51(8):999–1004. 30051623 10.1002/eat.22920

[31] Mang L, Garghan A, Grant J, Lacey H, Matthews R. An evaluation of efficacy and acceptability of a novel manualised JuniorLEAP group programme for compulsive exercise, for children and adolescents with anorexia nervosa, within an inpatient setting. Eat Weight Disord. 2021;26(2):591–7. 32232776 10.1007/s40519-020-00884-w

[32] Braun V, Clarke V. Using thematic analysis in psychology. Qual Res Psychol. 2006;3(2):77–101.

[33] Polit F, Beck Tatno C. Essentials of nursing research. 8 ed. ed. China: Wolters kluwer Health, Lippincott Williams & Wilkins; 2014.

[34] Davis C, Kennedy SH, Ravelski E, Dionne M. The role of physical activity in the development and maintenance of eating disorders. Psychol Med. 1994;24(4):957–67. 7892363 10.1017/s0033291700029044

[35] Davis C, Katzman DK, Kaptein S, Kirsh C, Brewer H, Kalmbach K, et al. The prevalence of high-level exercise in the eating disorders: etiological implications. Compr Psychiatry. 1997;38(6):321–6. 9406737 10.1016/s0010-440x(97)90927-5

[36] Wilson GT, Vitousek KM. Self-monitoring in the Assessment of Eating disorders. Psychol Assess. 1999;11(4):480–9.

[37] Bakland M, Rosenvinge JH, Wynn R, Sundgot-Borgen J, Fostervold Mathisen T, Liabo K, et al. Patients’ views on a new treatment for Bulimia nervosa and binge eating disorder combining physical exercise and dietary therapy (the PED-t). A qualitative study. Eat Disord. 2019;27(6):503–20. 30664397 10.1080/10640266.2018.1560847

[38] Brauhardt A, de Zwaan M, Hilbert A. The therapeutic process in psychological treatments for eating disorders: a systematic review. Int J Eat Disord. 2014;47(6):565–84. 24796817 10.1002/eat.22287

[39] Steinhausen HC. The outcome of anorexia nervosa in the 20th century. Am J Psychiatry. 2002;159(8):1284–93. 12153817 10.1176/appi.ajp.159.8.1284

[40] Dittmer N, Voderholzer U, von der Mühlen M, Marwitz M, Fumi M, Mönch C, et al. Specialized group intervention for compulsive exercise in inpatients with eating disorders: feasibility and preliminary outcomes. J Eat Disord. 2018;6:27. 30214803 10.1186/s40337-018-0200-8 PMC6131908

[41] Mathisen TF, Rosenvinge JH, Friborg O, Vrabel K, Bratland-Sanda S, Pettersen G, et al. Is physical exercise and dietary therapy a feasible alternative to cognitive behavior therapy in treatment of eating disorders? A randomized controlled trial of two group therapies. Int J Eat Disord. 2020;53(4):574–85. 31944339 10.1002/eat.23228 PMC7187559

[42] Byrne D. A worked example of Braun and Clarke’s approach to reflexive thematic analysis. Qual Quant. 2022;56(3):1391–412.

