# Supplementary material for: “To exercise sustainably” – Patients’ experiences of compulsive exercise in eating disorders and the Compulsive Exercise Activity Therapy (LEAP) as a treatment: a qualitative interview study
Source: J Eat Disord. 2024 Oct 1;12:151. doi: 10.1186/s40337-024-01115-8 (PMC11443868; doi:10.1186/s40337-024-01115-8)
Supplement: Supplementary file 1 — Supplementary Material 1: Additional file 1.docx contains “Additional materials to: “To exercise sustainably” – Patients’ experiences of compulsive exercise in eating disorders and LEAP as treatment: a qualitative interview study. The interview guide”, which is the semi-structured interview guide used in this study. [file 40337_2024_1115_MOESM1_ESM.docx]

**Additional materials to: "To exercise sustainably" – Patients’ experiences of compulsive exercise in eating disorders and the CompuLsive Exercise Activity TheraPy as treatment: a qualitative interview study.**

**The interview guide**

Can you to tell me when your eating disorder problems started?

How has your physical activity and exercise been through life? *(Suggested follow-up questions: how did your physical activity look like when the eating disorder started? Can you describe in more detail [e.g., type, duration, frequency]? What function has exercise had for you?)*

Can you describe what an ordinary day looks like for you?

Short facts: age, eating disorder diagnosis?

What made you want to participate in LEAP? What information did you get?

How did you experience the content of LEAP? *(If needed, give a short overview of the sessions)* How did you experience the content in relation to your own experiences (e.g., information, techniques, behavioral challenge)? *(If not physically active during LEAP; what was your behavioral challenge?)*

Did you experience something as missing? What did you acquire from the treatment?

What did participation in the LEAP treatment mean to you? *(If change occurred, when did it happen)*

How do you see your physical activity in the future? *(Suggested follow-up questions: what do you think about duration, frequency, type of activities? What do you want your physical activity to look like? What do you need to get there?)*

If you were to design an eating disorder treatment focusing compulsive exercise, what would it contain?

How would you experience physical activity in a group, led by a physiotherapist, as an addition to LEAP?

When, in time, did you participate in LEAP in relation to your ordinary treatment? *(Suggested follow-up questions: When would have been most preferable? What would have been the impact of earlier/later participation? Where are you currently in your treatment?)*

How did you experience LEAP as a supplement to your ordinary eating disorder treatment?

Which profession(-s) led your group?

Do you have something else to add?
